# Supplementary material for: Effects of CCL20/CCR6 Modulators in a T Cell Adoptive Transfer Model of Colitis
Source: Pharmaceuticals (Basel). 2025 Sep 4;18(9):1327. doi: 10.3390/ph18091327 (PMC12472583; doi:10.3390/ph18091327)
Supplement: Supplementary file 1 [file pharmaceuticals-18-01327-s001.zip › pharmaceuticals-3794668-supplementary.pdf]

Supplementary figures

Effects of CCL20/CCR6 Modulators in a T Cell Adoptive Transfer Model of Colitis

Marika Allodi, Lisa Flammini, Carmine Giorgio, Maria Grazia Martina, Francesca Barbieri, Vigilio Ballabeni, Elisabetta Barocelli, Marco Radi *, and Simona Bertoni *

Department of Food and Drug, University of Parma, Parco Area delle Scienze 27/a, 43124 Parma, Italy

* Correspondence: simona.bertoni@unipr.it (S.B.); marco.radi@unipr.it (M.R.).
Tel.: +39-0521905096 (S.B.)

Contents:

Figure S1. Representative flow cytometry analysis of murine lymphocytes

Figure S2. Representative flow cytometry FSC-SSC dot plot.

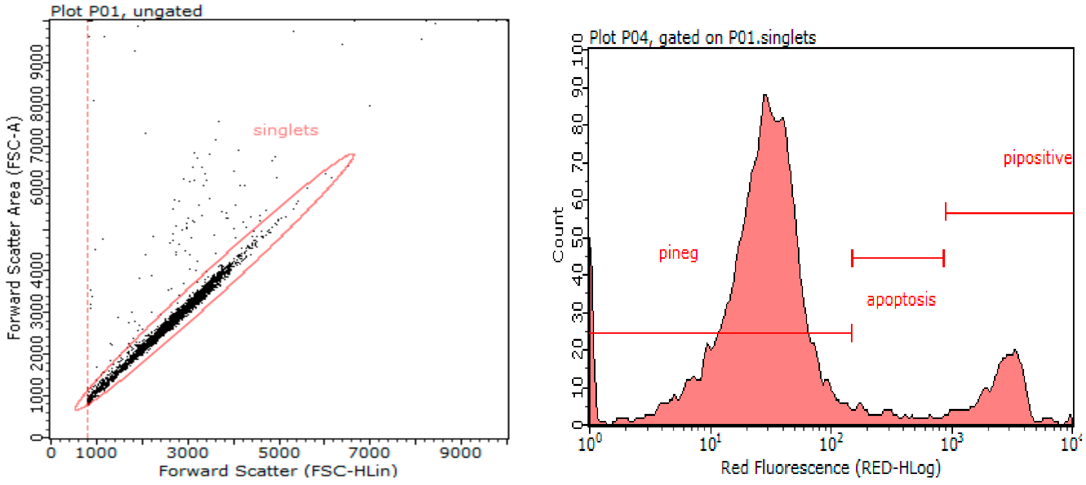

Figure S1:

Representative flow cytometry analysis of murine lymphocytes: (A) dot plot showing the singlet gating in FSC-A:FSC-H to exclude doublets; (B) histogram showing the viability threshold applied to singlets through PI assay.

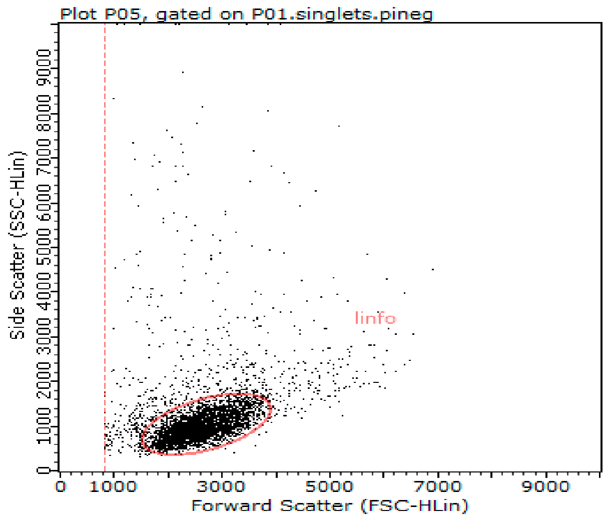

Figure S2:

Representative flow cytometry FSC-SSC dot plot showing MLN lymphocytes gated as events defined by FSC low: SSC low.
